# The outcomes of one-stage treatment for multiple knee ligament injuries combined with extensor apparatus rupture

**DOI:** 10.1186/s12891-020-03470-4

**Published:** 2020-07-09

**Authors:** Daohong Zhao, Zhongde Yang, Changsha Wu, Jia Zhong, Xizong Zhou, Jinghua Li, Yan Li, Yongsheng Lu, Duo Shen

**Affiliations:** 1 Department of Orthopaedics, The Second Affiliated Hospital of Kunming Medical University, No374, Dianmian road, Kunming, 650101 China; 2 Department of Orthopaedics, The People’s Hospital of WeiXin County, Zhaotong, China; 3 Department of Orthopaedics, The People’s Hospital of ZhenXiong County, Zhaotong, China; 4 Department of Orthopaedics, The People’s Hospital of XiShuangBanNa State, Jinghong, China; 5 Department of Orthopaedics, The People’s Hospital of YanJin County, Zhaotong, China; 6 Department of Orthopaedics, The Bone Special Hospital of JingHua Li, Zhaotong, China; 7 Department of Orthopaedics, The People’s Hospital of Dehong State, Mangshi, China; 8 Department of Orthopaedics, The People’s Hospital of NingLang County, Lijiang, China; 9 Department of Orthopaedics, The People’s Hospital of LongChuan County, Dehong, China

**Keywords:** Multiple knee ligament injuries, Extensor apparatus rupture

## Abstract

**Background:**

Multiple knee ligament injuries combined with extensor apparatus rupture are serious and complex knee injuries that are rare in clinical practice. The management is extremely challenging and controversial.

The aim of this study is to describe a patient collective with multiple knee ligament injuries combined with extensor apparatus injuries in detail and to report the mid-term outcomes of a one-stage surgical treatment regarding subjective outcome scores, complications, knee instability, and ROM.

**Methods:**

Eleven of 425 patients with multiple knee ligament injuries combined with extensor apparatus injuries admitted to our hospital were reviewed from July 2008 to May 2017. All patients underwent one-stage repair and reconstruction of multiple knee ligaments and extensor apparatus. The Lysholm knee score and the International Knee Documentation Committee (IKDC) score were adopted to evaluate the surgical effect preoperatively and at a minimum of 2 years’ follow-up. Clinical data, including range of motion and knee stability, were also recorded at the final follow-up.

**Results:**

Ten patients were followed up with a mean time of 40 (range, 24–60) months. At the last follow-up, 8 patients had joint flexion range of motion greater than or equal to120 degrees, 2 patients had joint flexion range of motion of 100–120 degrees, and 1 patient had active knee extension limitation of 5 degrees. Stress radiographs showed that the mean differences in posterior displacement were reduced from 10.8 ± 3.0 mm preoperatively to 2.0 ± 2.5 mm at the last follow-up. There were significant improvements in stress radiographs from pre- to postoperative states for all patients with multiple knee ligament injuries. The Lysholm score ranged from 85 to 96, with a mean of 92.1 (compared with 33 before surgery, *P* < 0.05). The final IKDC scores were A in 2 patients (20%), B in 7 (70%), and C in 1 (10%). Nine of the 10 patients (90%) returned to their former activity level.

**Conclusion:**

Multiple knee ligament injuries combined with extensor apparatus rupture are rare. Single-stage management of the repair and reconstruction of multiple knee ligaments and extensor apparatus with proper rehabilitation is an effective and reliable procedure to restore knee stability and function.

**Level of evidence:**

Level IV, therapeutic case series.

## Background

Multiple knee ligament injuries are not common in the clinic and mainly involve two or more groups of knee ligament structural injuries that can be combined with periarticular structural injuries or accompanied by varying degrees of nerve and vascular injuries or periarticular fractures [1–3]. Isolated injury of the extensor apparatus is mostly caused by trauma, and the main manifestations include quadriceps tendon injury, patellar fracture, patellar ligament injury, and avulsion fracture of the tibial tubercle [4, 5]. Multiple knee ligament injuries combined with extensor apparatus rupture are rare complex knee injuries that can lead to extreme instability of the knee, or, when combined with nerve and vascular injury, can threaten the affected limb. Treatment is extremely challenging, and improper treatment will seriously affect the knee function. Thus far, there have been few clinical reports on multiple knee ligament injuries combined with extensor apparatus rupture [6, 7]. Liu reported 15 patients with posterior knee dislocations associated with extensor apparatus ruptures in 2017; all patients underwent manual reduction and repair of extensor apparatus in the first stage and ligament reconstruction in the second stage, and good results were received. To our knowledge, there have not been any reports about one-stage procedures for these cases. In this series, we described a patient collective with multiple knee ligament injuries combined with extensor apparatus injuries in detail and reported the mid-term outcomes of a one-stage surgical treatment regarding subjective outcome scores, complications, knee instability, and range of motion (ROM). We hypothesized that complex multi-ligament injuries with extensor apparatus rupture can be treated with a one-stage procedure and that good function and knee stability could be obtained.

## Methods

### General information

**Fig. 1:**
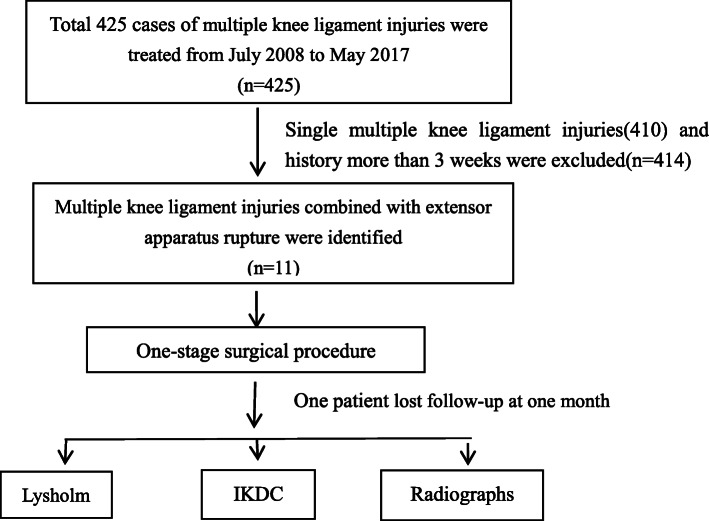
Flowchart of the study

**Fig. 2:**
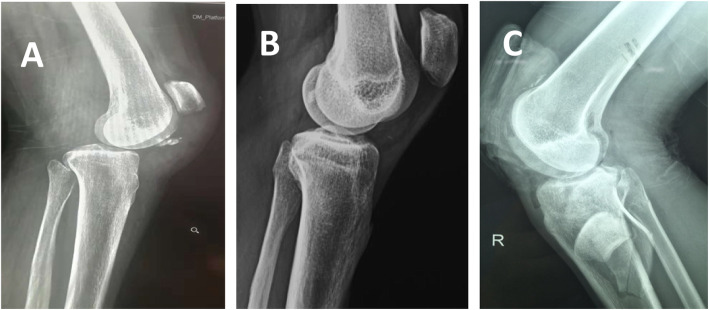
Different kinds of extensor apparatus injuries. **a** The inferior pole of a patellar avulsion fracture; **b** The mid-substance rupture of the patella tendon; **c** Open tibial plateau fracture with intra-osseous dislocation of the patella and quadriceps tendon rupture

A total of 11 patients admitted from July 2008 to June 2016 were detected, but only 10 patients were retrospectively analysed, including 8 males and 2 females. The average age was 26.5 years (range, 17–42). There were 6 cases of an affected right knee and 4 cases of an affected left knee. Nine cases were due to vehicle accidents, and 1 case was due to a fall; 1 case was an open injury, and 9 cases were closed injuries. In the same period, 425 cases of multiple knee ligament injuries were treated in our department, and the case selection criteria are shown in the flowchart (Fig. 1). Inclusion criteria: all patients with acute multiple knee ligament injuries combined with extensor apparatus rupture were included. Exclusion criteria: patients with more than 3 weeks of history or single or multiple knee ligament injuries or extensor apparatus rupture were excluded; patients with combined tibial plateau fracture or femoral condyle fracture were not excluded. Each patient was clearly diagnosed by preoperative X-ray (Fig. 2) and MRI. The specific injuries of each patient are shown in Table 1. All the data were approved by the Ethics Committee of the Second Affiliated Hospital of Kunming Medical University and were approved by the patients.

**Table 1:** General and surgical information of all patients

Patient No.	Sex	Age(y)	location	Cause	Close or open	Injury of extensor apparatus	Injury of ligament	Combined injury	Time to surgery (day)	Surgery time, min	Tension-reduced wire	Repaired or reconstructed ligament
Case 1	M	26	Left	Accident	Close	Patella tendon rupture	PCL + ACL + PLC	No	5	140	Used	PCL + PLC
Case 2	M	28	Right	Accident	Close	Patella fracture	PCL + ACL + PLC	No	5	120	No	PCL + PLC
Case 3	F	31	Left	Accident	Close	Patella fracture	PCL + ACL	No	7	130	No	PCL + ACL
Case 4	M	29	Right	Accident	Close	Quadriceps tendon rupture	PCL + ACL	Lateral femoral fracture	10	145	Used	PCL
Case 5	M	32	Right	Accident	Close	Patella tendon rupture	PCL + ACL + MCL	No	5	120	Used	PCL + MCL
Case 6	M	42	Left	Accident	Close	Patella tendon rupture	PCL + ACL + PLC	Peroneal nerve injury	8	125	Used	PCL + PLC
Case 7	F	34	Right	Accident	Close	Patella tendon rupture	PCL + ACL	No	7	100	Used	PCL + ACL
Case8	M	21	Right	Accident	Close	Patella tendon rupture	PCL + ACL + PLC	No	8	130	Used	PCL + PLC
Case9	M	25	Left	Fall	Close	Patella fracture	PCL + ACL	No	9	130	No	PCL + ACL
Case10	M	19	Right	Accident	Open	Quadriceps tendon rupture	PCL + ACL (avulsion fracture	Tibial palteau fracture	1	115	Used	PCL + ACL

*ACL* anterior cruciate ligament, *FCL* fibular collateral ligament, *MCL* medial collateral ligament, *PCL* posterior cruciate ligament, *PLC* posterolateral corner

To evaluate the stability of the knee, a stress radiograph obtained by use of a Telos stress device (Austin & Associates, Fallston, MD) was used both preoperatively and at the final follow-up. The Lysholm scoring system was used for follow-up assessment to document subjective symptoms. The standard knee ligament evaluation proposed by the International Knee Documentation Committee (IKDC) was also used. The IKDC score combines the assessment of both symptoms and signs. Each category is assigned an overall grade of A (normal), B (nearly normal), C (abnormal), or D (severely abnormal). A final grade of A, B, C, or D is determined by the lowest score in each category. Radiographs were obtained 3 and 6 months postoperatively, patellar position was assessed by the use of the Caton-Deschamps index according to the lateral radiographs, and the Kellgren-Lawrence score was used to assess the radiological osteoarthritis change. The *x*^2^ test was used to compare changes in the IKDC scores in groups A and B with those in groups C and D (normal or nearly normal *v* abnormal or severely abnormal) between the preoperative period and the final follow-up. The Mann-Whitney *U* test was applied for ranking continuous data (Lysholm scores). Statistical analysis was performed with SigmaStat software, version 2.0 (Systat Software, San Jose, CA). The results were statistically analysed with .05 as the significance level.

### Intra-operative findings and procedure

**Fig. 3:**
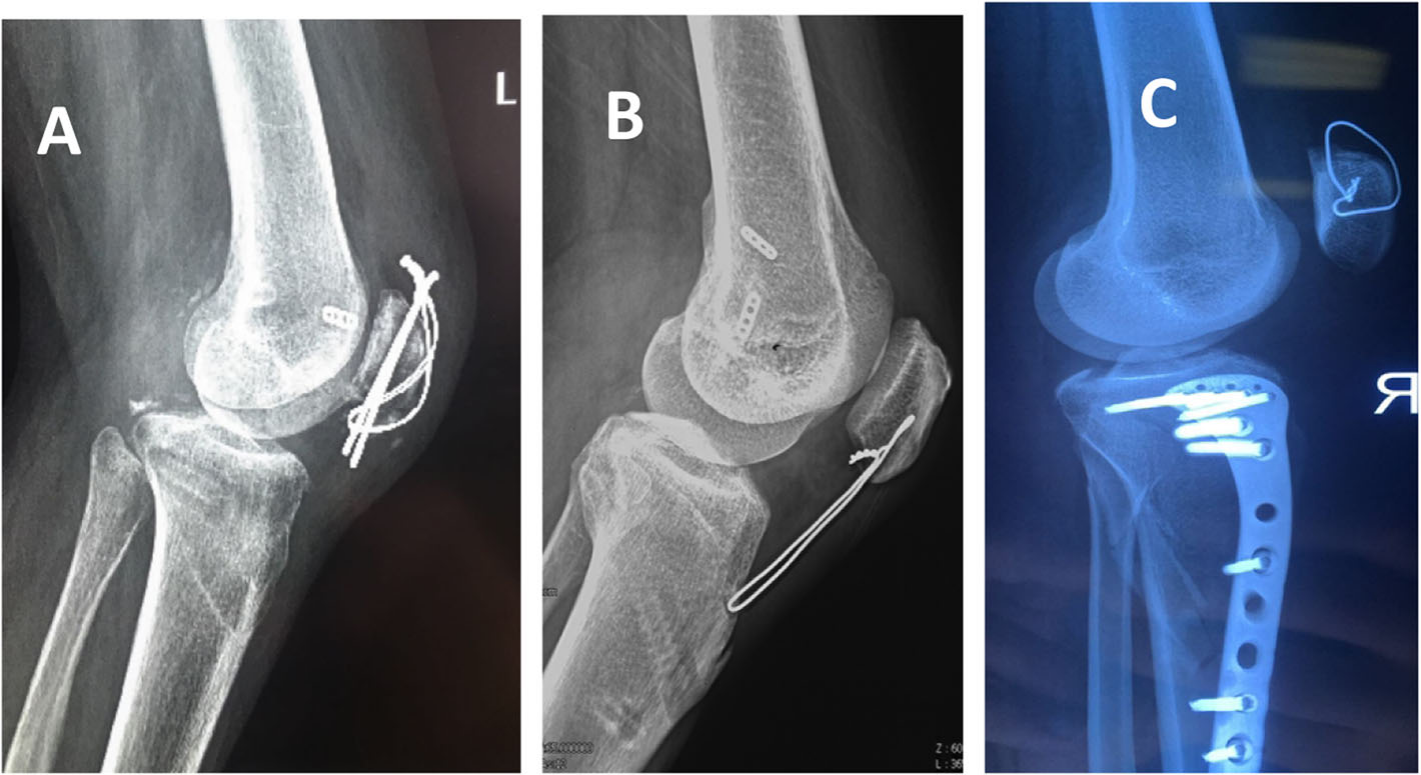
Different kinds of surgical methods for extensor apparatus injuries. **a** ORIF of a patellar fracture; **b** Repair and tension-reduced fixation for mid-substance rupture of the patellar tendon; **c** Repair and tension-reduced fixation of the superior pole of a patellar avulsion fracture and quadriceps tendon rupture

All patients underwent one-stage repair and reconstruction of multiple knee ligaments and extensor apparatus. First, the posterior and anterior cruciate ligaments (ACLs) were reconstructed using hamstring tendon under arthroscopy. We performed a selective procedure for ACL injuries; if the patient had PLC or medial collateral ligament (MCL) injuries, the ACL procedure was abandoned. The transplanted tendon was implanted, and then the medial and lateral structures were repaired or reconstructed, with repair or open reduction internal fixation (ORIF) of the damaged extensor apparatus by incision. If the extensor apparatus injuries were located in the patellar tendon mid-substance, it was important to maintain the normal patellar height after repair. Generally, we adjusted the patellar height to the level of intercondylar notch at knee 45° flexion. Finally, all the transplanted ligaments were tightened and fixed; generally, the extensor apparatus was repaired and fixed first, and the reconstructed cruciate ligaments were tightened and fixed last. Four patients with anterior and posterior cruciate ligament injuries combined with posterolateral structural injury were had the posterior cruciate ligament and posterolateral complex structure reconstructed using bilateral hamstring tendons. One patient with anterior and posterior cruciate ligament injuries and an MCL injury received posterior cruciate ligament reconstruction and MCL repair. Anterior and posterior cruciate ligament reconstructions were performed in 3 patients with anterior and posterior cruciate ligament injuries. One patient with an anterior and posterior cruciate ligament injury and a femoral epicondylar fracture was treated with posterior cruciate ligament reconstruction and femoral epicondylar fracture fixation. One patient with a tibial insertion avulsion fracture of the anterior and posterior cruciate ligaments and an open tibial plateau fracture (Schatzker II) was treated with internal fixation using suture and a metal plate. Three cases of patellar fracture were fixed by a tension-band technique with open reduction. Quadriceps tendon rupture in 2 cases was located in the upper pole of the patella, which was repaired by placing 3–4 anchors in the upper pole of the patella, suturing and repairing the quadriceps tendon by its suture, establishing a 2-mm transverse tunnel with K-wire in the middle of the patella, and guiding a 1-mm wire to the patella and crossing the quadriceps tendon for a tension-reduced fixation. Of the 5 patellar tendon injuries, 3 cases were located in the inferior pole of the patella; the surgical procedure was similar to that for the quadriceps tendon. Two cases were mid-substance ruptures, and after edge-to-edge suturing with non-absorbable suture, a 2-mm tunnel was established with a K-wire in the middle part of the patella and tibial tubercle, and a 1-mm wire was used to pass through the patella and tibial tubercle for a tension-reduced fixation (Fig. 3). One case with peroneal nerve palsy, oedema and compression of the peroneal nerve was confirmed, and full release was performed. More details are provided in Table 1.

### Rehabilitation

On the second day after the operation, ankle pump exercises with brace protection and straight leg elevation exercises were started. The knee extension position was fixed for 1 week. It is recommended to perform flexion and extension function exercises of the knee in the early stage and start flexion and extension function exercises 1 week after the operation. It is required to establish flexion to 90 degrees within 1 month after the operation and 120 degrees within 6 weeks. If flexion cannot reach 90 degrees within 1 month after the operation, manipulative relief is necessarily given to normal. All patients without concomitant fractures of the femur or tibia were partially weight bearing immediately postoperatively under the protection of a crutch, and full weight bearing was permitted at 1 month. Patients with fractures were weight-free within 1 month after surgery and protected by braces within 3 months after surgery. Patients with tension-reducing wires during knee extension should have them removed under local anaesthesia 2 months after surgery.

## Results

All the patients were examined by an independent observer (W-Z.D.) who was not involved in the surgery. The incidence rate of this concomitant pathology was nearly 2.5% (11/425). Ten patients were followed up, and one patient was lost to follow-up at 1 month after surgery. The mean follow-up time was 40 (range 24–60) months. There were no postoperative wound infections, thrombosis or other complications in any patient. In the 10 patients, the mean difference in posterior translation preoperatively and at the final follow-up, as measured by posterior stress radiography, was 10.8 ± 3.0 mm preoperatively, improving to 2.0 ± 2.5 mm at the last follow-up. The difference between the last follow-up evaluation value and the value before surgery was significant (*P* < .001). There were significant improvements in stress radiographs from pre- to postoperative states for all patients with ACL, MCL, and FCL/PLC injuries (Table 2). No patient was indicated for second surgery for any complaint of instability at follow-up.

**Table 2:** Pre- and postoperative stress radiographs for all patients with PCL, ACL, MCL and FCL/PLC injuries according to posterior, anterior, valgus and varus stress

Stress Radiograph	Preoperative	Postoperative	*P* Value
Posterior (PCL),*n* = 10(mean ± SD)	10.8 ± 3.0	2.0 ± 2.5	<.0001
Range	8.5 to 20.5	0.9 to 4.2	
95%CI	11.8 to 13.6	1.0 to 2.5	
Anterior (ACL),*n* = 10(mean ± SD)	8.2 ± 3.5	1.0 ± 1.2	<.0001
Range	6.5 to 14.5	0.6 to 3.2	
95%CI	7.5 to 9.8	0.6 to 1.5	
Valgus (MCL),*n* = 1	3.5	0.5	
Varus (FCL/PLC),*n* = 4(mean ± SD)	4.8 ± 0.5	0.5 ± 1.5	<.0001
Range	2.5 to 8.0	0.2 to 3.5	
95%CI	2.1 to 8.6	0.4 to 2.0	

*ACL* anterior cruciate ligament, *FCL* fibular collateral ligament; *MCL* medial collateral ligament, *PCL* posterior cruciate ligament; *PLC* posterolateral corner

Postoperative imaging review showed that the patella was in a good position, the Caton-Deschamps index showed that the patellar positions of all patients were normal according to the radiographs (Table 3), and all fractures had healed completely at the 6-month follow-up visit; there were no cases of wire breaks and no re-tears of the extensor apparatus. At the final follow-up visit, there was no obvious osteoarthritic changes according to the postoperative imaging; only two patients had mild radiological osteoarthritic changes compared to preoperation. The ROM of joint flexion was greater than 120 degrees in 8 patients and 100–120 degrees in 1 patient; 1 patient had an active knee extension limitation of 10 degrees. A patient with a peroneal nerve injury recovered completely in 6 months after surgery. Nine of the 10 patients had normal muscle strength of the quadriceps, and 1 patient had a mild weak force.

**Table 3:** The follow-up and evaluation of all patients

Patient No.	Last follow-up, mo	Range of motion	Lysholm scores#		IKDC rating		Kellgren-Lawrence score		Caton-Deschamps index	Complication
		Post-op	Pre-op	Post-op	Pre-op	Post-op	Pre-op	Post-op		
Case 1	36	0°-120°	32	92	C (abnormal)	B (nearly normal)	0	I	1.0	No
Case 2	48	0°-120°	39	94	D (severe abnormal)	B (nearly normal)	0	0	0.9	No
Case 3	60	0°-125°	30	95	C (abnormal)	A (normal)	0	0	1.2	No
Case 4	30	0°-110°	28	92	D (severe abnormal)	B (nearly normal)	0	0	1.1	No
Case 5	36	0°-120°	30	89	D (severe abnormal)	B (nearly normal)	0	0	1.0	No
Case6	36	0°-120°	31	96	C (abnormal)	B (nearly normal)	0	I	1.0	No
Case 7	24	0°-120°	35	94	D (severe abnormal)	B (nearly normal)	0	0	0.9	No
Case 8	40	0°-120°	35	94	D (severe abnormal)	A (normal)	0	0	0.9	No
Case9	42	0°-120°	42	90	D (severe abnormal)	B (nearly normal)	0	0	1.0	No
Case 10	48	5°-110°	28	85	D (severe abnormal)	C (abnormal)	0	0	1.0	No

#Paired *t* test, *p* < 0.001

The mean preoperative Lysholm score in the 10 patients was 33 (range, 28 to 42), and the mean postoperative Lysholm score was 92.1 (range, 85 to 96). Lysholm scores significantly differed between the preoperative evaluation and the final follow-up (*P* < .001).

At the final follow-up, the IKDC scores were observed to be normal (grade A) or nearly normal (grade B) in 9 patients (90%) and abnormal (grade C) in 1 patient (10%). Nine of the 10 patients (90%) returned to their former activity level (Table 3).

## Discussion

The most important result of this study is that single-stage management of repair and reconstruction of multiple knee ligaments and extensor apparatus injuries with proper rehabilitation is an effective and reliable procedure to restore knee stability and function. In this series, we performed a single-stage procedure including selective knee ligament reconstruction with PCL and PLC, PCL and MCL, or PCL and ACL and extensor apparatus fixation or repair with tension-reduced fixation. In some cases, we abandoned the ACL reconstruction procedure if the patient had PLC or MCL injuries because the ACL was not necessary to reconstruct multiple knee ligament injuries at the primary or secondary stage according to the literature [8, 9]. In our series, we performed the ACL procedure on only active and young patients. Reliable repair and reconstruction of damaged structures to restore stability allowed early mobilization; 8 of 10 patients achieved full ROM (0–120) at 3 months, only 1 patient experienced 20 degrees of flexion limitation, and 1 patient experienced 10 degrees of active extension limitation. According to the final follow-up evaluation, the Lysholm scores ranged from 85 to 96, with a mean of 92.1, which was significantly improved after operation. The IKDC scores were observed to be normal (grade A) or nearly normal (grade B) in 9 patients (90%) and abnormal (grade C) in 1 patient (10%). Nine of the 10 patients (90%) returned to their former activity level. One-stage procedure is beneficial to patients with stable joints, and a second surgery can be avoided, but a proficient technique and excellent rehabilitation programme are required.

**Fig. 4:**
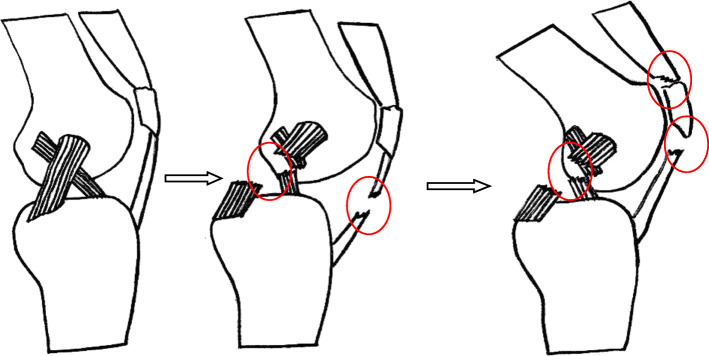
Schematic diagram showing the mechanism of multiple knee ligament injuries combined with different kinds of extensor apparatus injuries

Multiple ligament injuries of the knee joint represent serious high-energy soft tissue injuries. The knee joint is extremely unstable, often leading to dislocation of the knee joint and sometimes combined with nerve and blood vessel injury, which can result in a high disability rate and can endanger the affected limb or even the life of the patient with improper treatment [3, 10]. Based on the position of the tibia relative to the femur, in 1963, Kennedy [11] classified knee joint dislocation caused by multiple ligament injuries into five categories: anterior, posterior, internal, external, and rotational. Schenck [12] and Wascher [13] classified multiple knee ligament injuries into 5 types according to anatomy and injured structures. This classification has been widely used to date, but it is not a good guide for clinical treatment. Isolated extensor apparatus rupture is mostly caused by trauma. The main manifestations include quadriceps tendon injury, patellar fracture, patellar ligament injury, and avulsion fracture of the tibial tubercle. A possible mechanism for the concomitant injuries is that when posterior dislocation of knee joint occurs, the knee joint is located at different angles, and the forward femoral condyle will directly impact the force on the knee extension device. If the knee is located at nearly the extended position, it may cause a compression fracture in front of the tibial plateau or an avulsion fracture of the tibial tubercle. With the increase in the knee flexion angle, the force on the patellar ligament or the lower pole of the patella will cause tearing of the mid-substance or avulsion fracture of the lower pole of the patella, and the active contraction of the quadriceps will also cause tendon injury (Fig. 4). Among the cases in this group, 9 patients had obvious posterior dislocation of the knee joint, and the most common injury of extensor apparatus was mid-substance of the patellar ligament and avulsion fracture of the inferior pole of patella, similar to the 15 cases of knee joint dislocation combined with extensor apparatus injury reported by Liu [6] in 2017. In his cases, 11 of 15 were mid-substance injuries to the patellar ligament and avulsion fractures of the inferior pole of patella. Cenk [14] also reported a case of bilateral knee dislocation with patellar ligament injury in 2006. Multiple knee ligament injuries combined with quadriceps tendon rupture are rare; to our knowledge, have been no case reports to date. In this series, there were 2 patients with a femoral quadriceps tendon rupture located in the upper pole of the patella, all of which were high-energy traffic accident injuries, one patient with femoral condyle fracture, and one patient with an open tibial plateau fracture, which may have been caused by multiple injury factors.

Repair of the extensor apparatus rupture to restore its functions and allow early mobilization is widely recommended [15]. Its management depends on the location of the injuries. ORIF is best for patellar fractures and avulsion fractures, while suturing and repair are suitable for quadriceps tendon and patellar tendon ruptures to achieve reliable repair and allow early ROM. Traditionally, tension-reduced wires piercing the patella or tibial tuberosity are necessary for tendon rupture repair; some authors have also suggested a tendon augmentation technique using the hamstring tendon [7]. In this series, tension-reduced wires were used for the cases of quadriceps tendon or patellar tendon rupture, and most patients gained normal functional benefit from rehabilitation early with full ROM. However, due to the variety of multiple knee ligament injuries, the treatment modalities for these injuries are varying and controversial. Some published studies have supported a conservative approach, while increasing numbers of studies have favoured surgical treatment [8, 9]. Currently, reconstruction and repair of multiple knee ligaments to restore knee stability are widely recommended [16]. Regarding the management of the multiple knee ligament injuries combined with extensor apparatus rupture, there have been few studies published, Liu et al. [6] reported a series of 15 cases with a two-stage procedure; the primary management was reduction and repair of the extensor apparatus, and multiple ligament reconstruction was followed for at least 6 weeks. Most of patients achieved normal ROM and knee stability. O’Malley et al. [7] reported 2 cases of extensor mechanism disruption in knee dislocation; one patient had a patellar tendon rupture with knee dislocation and received a single-stage procedure, including reconstruction of the ACL, medial and lateral meniscal repairs, MCL repair, and patellar tendon repair with allograft augmentation, while the other ad the same pathology and received a two-stage procedure involving opening of the lateral meniscus, MCL and patellar tendon repair using allograft augmentation. ACL, PCL, and MCL reconstruction occurred at the second stage, and both patients achieved good outcomes with active rehabilitation.

Neurovascular injury in multiple knee ligament injuries is common; the mean incidence of popliteal artery insults is 30% (range 17–55%) [17]. A common peroneal nerve palsy complicates knee dislocations in 5 to 40% of patients [18]. There were no neurovascular injuries in this series; the main probable reason is that most patients had posterior dislocation, and when it resulted in extensor apparatus rupture, the main energy was absorbed through the anterior damaged structure, and the possibility of posterior neurovascular injuries was decreased. Only 1 patient had peroneal nerve palsy; after exploration and release, the symptoms had disappeared at the 6-month follow-up.

The most important purpose of this study is that it is the first to report different types of extensor apparatus rupture combined with multiple knee ligament injuries and to evaluate the clinical outcomes of single-stage management. However, admittedly, this study has some limitations. First, this was a retrospective study. Second, due to the extremely low incidence of extensor apparatus rupture combined with multiple knee ligament injuries, this study involved only a small population. Third, the follow-up time was relatively short, and longer-term evaluations are required to evaluate the long-term clinical outcome.

## Conclusion

This present study demonstrated that multiple knee ligament injuries combined with extensor apparatus rupture are rare and are mostly associated with posterior knee dislocation and patellar tendon rupture. Good clinical results can be achieved without any complications related to the single-stage procedure.

## Data Availability

Data associated with this study is retained at a central repository at the Orthopaedic Department, the Second Affiliated Hospital of Kun Ming Medical University. If there are any questions, please contact the corresponding author.

## Abbreviations

ACL: anterior cruciate ligament
PCL: posterior cruciate ligament
PLC: posterior lateral complex
MCL: medial collateral ligament
